# Network crafting, goal attainment, and work-to-family facilitation among hotel employees: the mediating roles of positive affect and information exchange

**DOI:** 10.3389/fpsyg.2023.1279250

**Published:** 2023-12-05

**Authors:** Hongshuo Zhang, Jiakun Liu, Huatian Wang, Kongqi Li

**Affiliations:** ^1^Faculty of International Tourism and Management, City University of Macau, Macao, Macao SAR, China; ^2^School of Economics and Management, Shandong Youth University of Political Science, Jinan, China; ^3^Department of Psychology, Lingnan University, Hong Kong, Hong Kong SAR, China

**Keywords:** network crafting, goal attainment, work-to-family facilitation, positive affect, information exchange

## Abstract

**Background:**

The hospitality industry is experiencing new developmental opportunities after the coronavirus pandemic, such as the expansion of digital presence, the introduction of wellness offerings to cater to health-conscious guests, and a growing focus on local and sustainable tourism. However, despite these positive changes, we still lack knowledge on how hospitality workers can proactively adjust their work conditions to excel in their professional domain while also flourishing in their family domain. Thus, the current study proposed and examined how network crafting behaviors can have positive effects on hotel employees’ work goal attainment and work-to-family facilitation. Based on the affectivity theories and the social cognitive theory, we examined the mediating roles of positive affect and information exchange on the relationship between network crafting behaviors and work goal attainment and work-to-family facilitation.

**Methods:**

We collected data from three 5-star hotels in Jinan, China. We sent out the surveys in three waves to avoid the common method bias. We obtained 199 valid responses in total in three waves and entered them into the data analysis. Structural equation modeling was conducted to examine our hypotheses.

**Results:**

We found that network crafting was positively related to hotel employees’ work goal attainment and work-to-family facilitation. We also confirmed the mediating roles of positive affect and information exchange in this relationship.

**Conclusion:**

We revealed a dual process of network crafting – that is, a positive affective process and an information exchange process. We contribute to the social network and networking literature by highlighting an optimization-oriented networking strategy, rather than one simply maximizing networks. We enrich the work-family enrichment literature by suggesting an effective behavioral strategy that can transmit the resources and gains from one domain to the other domain.

## Introduction

The hospitality industry has been recognized as one of the most labor-intensive (i.e., physical and emotional labor) industries involving high work overload and inflexible work schedules (Xu and Chris Cao, 2019; Liu et al., 2022). Hotel employees face a growing array of challenges in both their work and family domains in today’s environment. For example, employees in guest-facing roles such as front desk staff, concierge, and sales representatives are frequently required to engage in extensive networking behaviors to establish and maintain relationships with a diverse customer base (Teng, 2019). Besides, in order to achieve career advancement and improve work outcomes, hotel employees must actively cultivate connections with industry professionals, mentors, and leaders. In addition to the demands within the work domain, hotel employees also encounter significant obstacles in achieving work-family balance. The long and irregular working hours, often involving shifts, make it difficult for them to strike a healthy work-life equilibrium and spend quality time with their families (Lee and Ok, 2015). Moreover, hotel employees are expected to provide exceptional customer service and manage emotional labor, which can be emotionally draining and lead to exhaustion that may spill over into their family lives (Xu and Chris Cao, 2019). Given the nature of intensive interactions with various individuals inherent to hotel work, it is crucial for researchers and managers to identify effective strategies that enable hotel employees to attain their work goals while also facilitating a healthy work-to-family balance.

Previous studies revealed many factors that can contribute to the improvement of hotel employees’ work and non-work outcomes, ranging from the individual level (e.g., core self-evaluations, job crafting, informal learning; Liu et al., 2022), team level (e.g., team cohesion and trust; Han et al., 2016; Hussain et al., 2016), to the organizational level (e.g., inclusive HR practices, managerial support for work-family balance; Liu et al., 2022). However, we found that few studies took a social network perspective to understand the process by which social interactions/social capital facilitate hotel employees’ work and non-work outcomes. Although a small handful of studies uncovered the beneficial role of social networking and networking ties in the hospitality setting, we found that these studies largely focused on organizational-level outcomes (e.g., entrepreneurial orientation, organizational learning) but with little attention on employee-level outcomes (Avci, 2020; Tajeddini et al., 2020). More importantly, we found that previous studies mainly took the expansive-oriented networking perspective to understand the networking process. That is, individuals or actors make efforts to increase the number of contacts and maximize the benefits of networks. However, recent scholars (van Gool, 2022; Wang et al., 2023) argue that networks include both benefits and costs [i.e., time and energy invested in social interactions and interacting with difficult or dissonant colleagues (Brennecke, 2020)]. One should optimize these benefits and costs within the network (based on his/her personal needs and goals) rather than simply expanding the network. Thus, an optimization-oriented networking strategy seems a more appropriate approach to gain and maintain useful social capital and create a person-network fit.

Based on previous networking studies (Wolff and Moser, 2009; Porter and Woo, 2015) and the job crafting literature (Wrzesniewski and Dutton, 2001; Tims et al., 2012), we propose that network crafting, referring to an optimization-oriented networking strategy where employees proactively seek resources and optimize demands in the network based on their personal needs and goals (van Gool, 2022), may help to facilitate hotel employees’ work and non-work outcomes. In the current study, we consider work goal attainment as a work-related outcome and work-to-family facilitation as a non-work-related outcome. We aimed to examine the beneficial role of network crafting on hotel employees’ work goal attainment and work-to-family facilitation. Further, we aimed to deepen the understanding of why and how network crafting behaviors can have positive impacts on hotel employees’ work and non-work outcomes. Therefore, based on the broaden-and-build theory (Fredrickson, 2004) and the social cognitive theory (Bandura, 2001), we further examined the mediating roles of positive affect (an affective mediator) and information exchange (a cognitive mediator) on the relationship between network crafting and hotel employees’ work goal attainment and work-to-family facilitation. The reasoning is that via network crafting, employees may gain a positive affective process, which can broaden thoughts and actions and build personal resources. Also, via network crafting, employees may initiate a social learning process where they can exchange, share, and learn useful information and knowledge from others. Consequently, the positive affective process and social learning process can result in work goal completion and work-to-family facilitation.

**Figure 1:**
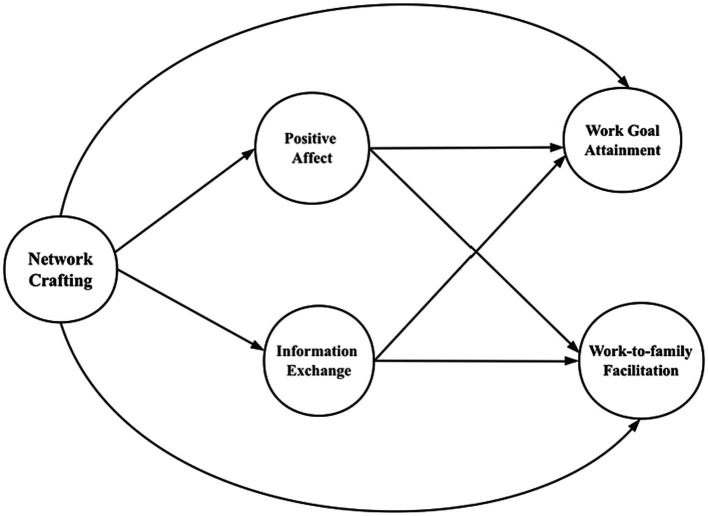
Conceptual model.

This study aims to contribute to literature in several ways. First, we add to the job crafting literature (Wrzesniewski and Dutton, 2001; Tims et al., 2012). Since the concept of network crafting was derived from job crafting, we enrich this line of research by suggesting that in addition to crafting general jobs, crafting one’s professional networks is also a meaningful way to maintain a motivational process and reduce the health impairment process in the workplace. Second, we consider network crafting as an optimization-oriented networking strategy, and thus we contribute to the networking literature (Carpenter et al., 2012; Porter and Woo, 2015) by highlighting that an effective social interaction process and an access to social capital necessitate how one can balance the constellation of resources and demands in his/her network. We underscore that network crafting is a more self-customized networking process where employees adjust different networking behaviors based on personal situations, interests, and goals, rather than blindly expanding the network. Third, we add to the work-family enrichment studies (Greenhaus et al., 2006; Liu et al., 2022), since one of the outcomes of network crafting is work-to-family facilitation. Examining a dual process of network crafting, we reveal that network crafting can activate a positive affect process and an information exchange process, which can transmit the gains (e.g., skills, affective, capital, or efficiency) from the work domain to the family domain (Wayne et al., 2007). Finally, we contribute to the hospitality management literature (Xu and Chris Cao, 2019; Liu et al., 2022) by suggesting a new work strategy for hotel employees – network crafting. We indicate that network crafting can be effective in the improvement of hotel employees work and non-work outcomes. Purposefully crafting professional networks can help hotel employees sustain a highly motivational process at work and enhance the functioning of family life. See Figure 1 for our conceptual model.

## Literature review and hypothesis development

### Broaden-and-build and social cognitive theories

In the current study, we use broaden-and-build and social cognitive theories (Bandura, 1989; Fredrickson, 2004) to understand how network crafting behaviors facilitate employee work/nonwork outcomes through positive affect and information exchange. The broaden-and-build theory suggests that positive emotions can serve two primary functions: broadening function, which broadens individuals’ thought-action repertoire, and building function, which can help the development of enduring personal resources, such as social support networks, coping strategies, and a positive self-concept (Fredrickson, 2004). Therefore, it is argued that those who actively craft their networks are able to gain positive affect. These positive emotions can broaden one’s thoughts and actions and enhance their personal resources. As a result, employees are better able to complete work tasks and goals in the work domain. Furthermore, these positive gains can spillover to the family domain and bring beneficial family outcomes as well.

The social cognitive theory (Bandura, 1989) posits that individuals can learn and develop through a dynamic interaction between personal factors, environmental influences, and their own behaviors. Thus, in addition to an affective process, we argue that network crafting can also stimulate a social learning process. Through network crafting, individuals have the opportunity to interact with others, exchange valuable information, and acquire knowledge. As a result, this information exchange enables employees to effectively manage their work goals and tasks. Furthermore, the knowledge gained through networking can also have positive implications in the family domain. Employees can leverage the information acquired from their networks to enhance their effectiveness in managing family-related responsibilities and challenges.

### Network crafting

The concept of network crafting is derived from the job crafting literature (Wrzesniewski and Dutton, 2001; Tims et al., 2012), which was first proposed by van Gool (2022). van Gool (2022) proposed that network crafting is an optimization-oriented networking strategy where employees proactively seek resources and optimize demands in the network based on their personal needs and goals. According to the job demands-resources model (Bakker and Demerouti, 2017), resources refer to those functional and motivational aspects that can help employees achieve work goals, solve problems, and stimulate personal growth, learning, and development, while job demands refer to some aspects of the job that require sustained physical and/or psychological effort or skills, which will result in certain physiological and/or psychological costs (Bakker and Demerouti, 2017). Therefore, in one’s professional network, resources can be colleagues’ expertise and knowledge, emotional support, and/or supervisors’ coaching and feedback; while demands can be time and energy costs in social interaction processes, and some incidents and persons that make employees feel angry, anxious, or exhausted. Network crafting aims to balance the constellation of resources and demands in the network and to increase person-network fit. van Gool (2022) further pointed out that network crafting is a form of proactive behavior and resembles relational crafting (one of the aspects of job crafting). But network crafting has a narrower scope than job crafting (i.e., focusing on professional networks and involving tailored networking behaviors).

Nevertheless, network crafting differs from traditional networking behaviors (Forret and Dougherty, 2004; Porter and Woo, 2015). Although network crafting involves networking-related behaviors, such as using, building, and maintaining relationships with others (Wolff and Moser, 2010; Wolff et al., 2011), network crafting manifests more than that. According to the job crafting literature (Wrzesniewski and Dutton, 2001; Tims et al., 2012) and the study of van Gool (2022), network crafting involves a cognitive reflection process (e.g., thinking about whom to network and how to network) before engaging in specific networking behaviors. Network crafting emphasizes strategically analyzing and adjusting the network as a whole instead of fixing or improving single relationships. In line with the notion of job crafting (Demerouti and Peeters, 2018), network crafting is concerning smartly optimizing network conditions based on individualized needs and goals rather than blindly expanding contacts.

Previous studies have shown consistent evidence of the beneficial effect of employee (job) crafting behaviors on various work outcomes, such as work performance, job satisfaction, work meaningfulness, work engagement, and organizational commitment [see meta-analyses (Rudolph et al., 2017; Lichtenthaler and Fischbach, 2019; Zhang and Parker, 2019)]. In the study of van Gool (2022), network crafting was found to positively relate to employee creativity and work engagement. Also, the study found that problem solving demands may foster employee network crafting behaviors and that role ambiguity may impede the emergence of network crafting behaviors (van Gool, 2022). Wang et al. (2023) conducted an intervention study and found that network crafting increased participants’ career autonomy, perceived marketability, and ego-network diversity (Wang et al., 2023).

### Network crafting and work goal attainment

While van Gool (2022) and Wang et al. (2023) revealed several network and career outcomes of network crafting (van Gool, 2022; Wang et al., 2023), work task-related outcomes and non-work outcomes are still less known. In the current study, we aim to extend the understanding of the outcomes of network crafting. We examined two outcomes: work goal attainment (work outcomes) and work-to-family facilitation (non-work outcomes). We hope to provide insights into whether and how network crafting can help employees to attain daily task goals and facilitate their family life.

Work goal attainment refers to the extent to which a work-related target has been achieved or completed via time and effort (Gollwitzer and Oettingen, 2001). Based on the social capital and social resources perspectives (Reagans and Zuckerman, 2001), one’s network is helpful to facilitate goal attainment, because social networks can provide individuals with task-related information and other invisible resources (Zhou et al., 2009; Liu et al., 2017). These can help employees deal with multiple challenges at work and keep track of the goal implementation process. The social capital view (Reagans and Zuckerman, 2001; Kilduff and Brass, 2010) further denotes that social relationships are resources/assets for individuals and can potentially lead to the development of human capital. Social networks (especially strong ties) can provide individuals with emotional support and trust (Perry-Smith and Shalley, 2014), which can further increase employees’ self-efficacy and resilience (Smith et al., 2008). Further, research found that weak ties can spark creativity, as they can provide non-overlapped information and some unique perspectives (Baer, 2010). Thus, actively using and shaping networks can gain valuable interpersonal resources and facilitate goal attainment. Previous networking studies showed that engaging in networking-related behaviors was positively related to work performance, professional advancement, and favorable performance appraisals from supervisors (Porter and Woo, 2015; Liu et al., 2017). Therefore, we argue that those who engage in network crafting actions can successfully get access to the necessary resources they need for task completion and actively optimize the challenges and difficulties in the social interaction process that may impede goal attainment. Thus:

*H1. Network crafting is positively related to work goal attainment*.

### Network crafting and work-to-family facilitation

Work-to-family facilitation refers to the extent to which an individual’s engagement in work domain provides gains (i.e., development, affective, capital, or efficiency) which contribute to enhanced functioning of family domain (Wayne et al., 2007). The work-family enrichment theories (e.g., the work-home resource model, Ten Brummelhuis and Bakker, 2012; emotion spill-over perspective, Wayne et al., 2007) suggest that resources gained in one domain (i.e., work) can be used, sustained, and developed in the other domain (i.e., home). These positive emotions experienced in the work domain can accelerate the momentary expansion of an employee’s thought-action inventory to the family domain. The work-family enrichment model particularly highlights that personal characteristics, such as positive affectivity, self-efficacy, and work identity are positively related to work-to-family facilitation (Wayne et al., 2007; Shockley and Singla, 2011). This is because these personal characteristics can promote one’s positivity, enabling individuals to more readily experience positive emotional states, and seek positive development developmental experiences (Wayne et al., 2007). Studies also found that some proactive actions, such as organizational citizenship behavior (Reizer et al., 2020), innovative work behavior (Mishra et al., 2019), job crafting (Rastogi and Chaudhary, 2018), and networking (Baumeler et al., 2018), can link to higher work-to-family facilitation. The reason is that, based on the work-family enrichment model (Baumeler et al., 2018), these proactive behaviors can help to build up social resources (e.g., colleague and supervisor support) and result in a positive return (e.g., information, energy, positive affect) (Baumeler et al., 2018). Therefore, we argue that by engaging in network crafting actions, employees may obtain positive psychological states and valuable social resources. When employees craft their professional network in the work domain, such as reaching out to a new colleague to learn a novel skill and/or receiving some support and constructive suggestions from existing contacts, this not only can make them feel inspired, enriched, and/or meaningful but also may trigger employees to apply what they learned from the work domain to their family domain. As a result, network crafting can contribute to the development of employees’ family life and facilitate family well-being. A study of Demerouti et al. (2019) found that those who actively crafted their job resources and demands in the work domain were also active in crafting their home resources and demands as well (Demerouti et al., 2019). Taking together:

*H2. Network crafting is positively related to work-to-family facilitation*.

### Positive affect as an affective mediator

Positive affect refers to the extent to which an individual subjectively experiences positive moods such as joy, interest, and satisfaction (Miller, 2011). Positive affect can occur when the individual is satisfied with the present state of affairs or a source of threat has been avoided (Pressman et al., 2019). The affectivity related theories (e.g., the broaden-and-build theory; Fredrickson, 2004) posit that specific work events or activities have an impact on the arousal of affective reactions and that positive emotions can broaden one’s awareness and spark novel, exploratory thoughts and actions. This broadened behavioral repertoire can help employees to build up useful skills and psychological resources, which can improve various work/non-work outcomes (Fredrickson, 2004). In addition, the emotion spillover view denotes that the positive affect gained from a network crafting process in the work domain can be spilled over to the family domain and improve the functioning of the family life (Wayne et al., 2007). Therefore, we argue that network crafting, as a positive, network-related activity, may activate employees’ positive affect at work. This is because network crafting enables employees to gain useful resources and/or emotional support from networks. Subsequently, with increased positive affect, employees are able to have a higher level of motivation and energy to deal with work tasks and attain the work goals they set.

Previous empirical evidence confirmed the meditating role of one’s positive affect between employee work-related activities and various work/non-work outcomes: Baranik and Eby (2016) found that employee organizational citizenship behaviors could negatively relate to burnout and positively relate to life satisfaction through the mediating role of positive affect (Baranik and Eby, 2016). Meyers and van Woerkom (2017) revealed that employees used their personal strengths at work can increase positive affect, which in turn, improved their work well-being (Meyers and van Woerkom, 2017). Others found that employee networking behaviors can link to a higher level of career satisfaction and work-to-life enrichment through increasing positive affect (Baumeler et al., 2018; Volmer and Wolff, 2018). The meta-analysis on positive affect showed that positive affect can link to greater social, psychological, and physical resources, and in turn, can predict more health behaviors and even less immune and cardiovascular diseases (Pressman et al., 2019). Another meta-analysis on the role of affectivity and job satisfaction showed that positive affect can predict 10–25% of variance in job satisfaction (Connolly and Viswesvaran, 2000). Therefore, based on the affectivity theories and previous evidence, we hypothesize:

*H3. Positive affect mediates the positive relationship between network crafting and (a) work goal attainment and (b) work-to-family facilitation*.

### Information exchange as a cognitive mediator

Information exchange is defined as the extent to which employees share and exchange work-related information, ideas and knowledge (Gkorezis and Bellou, 2016). Based on the social cognitive theory (Bandura, 2001), learning occurs in a social context with a dynamic and reciprocal interaction of the person, environment, and behavior. Bandura (2001) further emphasizes that by social learning and observing, individuals can increase self-efficacy and self-control (Bandura, 2001). Following this logic, information exchange can effectively boost a social learning process and may activate learning motivation and behaviors. The work-family enrichment theories (Wayne et al., 2007; Shockley and Singla, 2011; Ten Brummelhuis and Bakker, 2012) indicate that one receives gains, such as cognitive gains (new skills and perspectives), capital gains (economic, social, and health assets), and affective gains (positive emotion) in one domain can enhance the functioning of the other domain. Therefore, we argue that network crafting may first facilitate information exchange with contacts. This is because employees may receive valuable information from contacts but also bring out their own information that contacts may need (this reciprocal process is manifested in a network crafting process). Subsequently, when employees exchange information with their contacts, they will be able to use relevant informational cues to solve work-related problems and achieve work goals. Also, when employees receive valuable information, knowledge, or unique insights from different contacts, this may also trigger them to think about how it can be used in other life domains (e.g., the family domain).

Previous empirical evidence showed the mediating role of information exchange between social capital and learning outcomes (Wu, 2008; Wei and Ju, 2010; Gong et al., 2012). For example, studies found that effective social interactions can facilitate information exchange and sharing, which in turn, increase knowledge creation and even organizational competitiveness (Wu, 2008; Wei and Ju, 2010). Gong et al. (2012) found that employees with high proactivity and goal orientation can boost creative performance via information exchange (Gong et al., 2012). Therefore, based on the social cognitive theory and previous evidence, we hypothesize:

*H4. Information exchange mediates the positive relationship between network crafting and (a) work goal attainment and (b) work-to-family facilitation*.

## Methods

### Participants and procedures

We collected data from three hotels with five stars (i.e., Sheraton, Hilton, and Kempinski) in Jinan, China. The authors of this study used their personal networks to establish the collaboration for this study. That is, we used a convenient sampling approach to collect data. Once we obtained the agreement from the leaders of the hotels, we started to send out our surveys. We used Wenjuanxing, an online survey platform which is free and widely used in China, to develop the surveys. The inclusion criteria are that participants have a full-time contract with one of the three hotels. Part-time employees or internship students were excluded from this investigation. Before the official start of the survey, we sent a link to participants in which we included a general introduction of the study and the consent form we asked participants to agree on. We indicated in the consent form that the survey will be completely voluntary, confidential, and anonymous. The surveys were sent between February and March in 2023. Each survey has a two-week interval to reduce the impact of the common method bias (Antonakis et al., 2010). In the first wave (T1), we measured the independent variable (network crafting). After 1 week, we measured two mediator variables (positive affect and information exchange) at T2. After another 1 week, we measured two outcome variables (work goal attainment and work-to-family facilitation) at T3. To match participants’ data in each wave, we asked participants to create a unique identification code (and they had to remember it). We received 260 responses at T1, 251 responses at T2, and 199 responses at T3. After matching data, we obtained 199 valid participants that filled in the questionnaires at all three time points.

### Measures

We sent out the questionnaires in the Chinese language as our participants were all Chinese. To ensure content validity, we followed the back-translation procedure (Brislin, 1976). Specifically, we translated the items into Chinese first. The items were then translated literally back into English by a second expert linguist. To ensure that the back translation is accurate and not misleading, we compared it to the source text.

*Network crafting* (T1) was measured with five items developed by van Gool (2022). The five-point Likert scale was used (1 = strongly disagree, 5 = strongly agree). The items were: “I expand my relational network to effectively achieve my work goals”; “I increase the amount of communication I have with co-workers to get my job done”; “I increase the extent to which I deal with other people including co-workers and clients/customers”; “I deliberately reduce some interactions with some co-workers to get my job done”; “I deliberately reduce some interactions with some clients/customers to get my job done effectively.” The internal consistency showed good fit (*α* = 0.856).

*Positive affect* (T2) was measured with four items developed by Liu et al. (2020). The five-point Likert scale was used (1 = never, 5 = always). An example item was “I feel upset.” The internal consistency showed good fit (*α* = 0.886).

*Information exchange* (T2) was measured with four items developed by Gkorezis and Bellou (2016). A seven-point Likert scale was used (1 = very unlikely, 7 = very likely). A sample item was “I share information and learn from my colleagues.” The internal consistency showed excellent fit (*α* = 0.922).

*Work goal attainment* (T3) was self-evaluated by participants from 0% (no attainment) to 100% (complete attainment) for daily work tasks (see Grant et al., 2009). We transformed it into a 10 scale (i.e., 0 for no attainment, 10 for complete attainment) to align with the scaling of the measurements of other constructs. We also controlled for goal difficulty and goal time spending, which we explained below.

*Work-to-family facilitation* (T3) was measured with four items developed by Holbrook (2005). A seven-point Likert scale was used (1 = strongly disagree, 7 = strongly agree). A sample item was “Talking with someone at work helps me deal with challenges at home.” The internal consistency showed excellent fit (*α* = 0.901).

### Control variables

We included age, gender, tenure, and educational degree as controls because these demographic variables may influence individual cognitions and behaviors. Besides, Grant et al. (2009) suggested that goal attainment can be influenced by the extent of difficulty of the goal and how much time individuals spend in achieving the goal (also see Wang et al., 2022). Therefore, we measured *goal difficulty* with a single item “how difficult do you feel when you pursue the completion of daily work task?,” ranging from 1 = very easy to 4 = very difficult (Grant et al., 2009). *Goal time spending* was measured with a single item, ranging from 1 = less than 1 h; 2 = 1 to 2 h; 3 = 2 to 3 h; 4 = 3 to 4 h; 5 = 4 to 5 h; 6 = more than 5 h.

### Statistical approach

We performed the structural equation modeling (SEM) approach using Mplus to test our hypotheses. SEM has its benefits as it can examine latent variables, generate higher-order constructs, and well manage measurement error, which is one of the greatest limitations in most regression approaches. We will predict the measurement model by evaluating model fit indices such as chi-square (*χ*^2^), degree of freedom (df), CFI, TLI, SRMR, and RMSEA. We will conduct chi-square difference tests to compare different measurement models and confirm the construct discriminant validity. We will predict the structural model by reporting R square, Beta coefficient and standard error of each path regression, t-statistics, and value of *p*.

### Common methods bias test

Since the data were all self-reported, we did the common methods bias check using the latent method factor test using Mplus (Podsakoff et al., 2011), as Harman’s one-factor technique got some criticism for its insufficient sensitivity to detect moderate or small levels of common method variance effects (Malhotra et al., 2006). Our results indicated that the unconstrained model fit was *χ*^2^ = 291.755, df = 148; while the constrained model fit was *χ*^2^ = 308.011, df = 159. The Chi-square difference test showed no significance: Δ*χ*^2^ = 16.256, Δdf = 11, *p* = 0.132. This indicated that there was no significant “method” factor among the constructs. If we looked at the shared variance of the potential “method” factor, the equal-constrained model further showed that the “method” factor only shared a variance of 0.024 (i.e., 2.4% of shared variance). Thus, we can conclude that the common methods bias would not be an issue in the current study.

## Results

### Descriptive statistics

The descriptive analysis showed that the average age of employees was 25.55 (SD = 6.83). The average working tenure was 2 years (SD = 1.09). 72.4% were females and 27.6% were males. 82.4% of them had obtained a university degree or above.

The results of correlation analysis were presented in Table 1. We found that network crafting positively correlated to work goal attainment (*r* = 0.59**); network crafting positively correlated to work-to-family facilitation (*r* = 0.66*); network crafting positively correlated to positive affect (*r* = 0.45**) and information exchange (*r* = 0.62**). We also found that tenure significantly correlated to network crafting, work goal attainment, and work-to-family facilitation. That is why we considered it as the control in our analysis.

**Table 1:** Means, SD, and correlations among studied variables.

	**Mean**	**SD**	**1**	**2**	**3**	**4**	**5**	**6**	**7**	**8**	**9**	**10**	**11**
**1. Age**	25.55	6.83											
**2. Gender**	1.28	0.45	−0.07										
**3. Edu degree**	4.75	0.60	0.27**	−0.02									
**4. Tenure**	1.86	1.09	0.67**	−0.04	0.26**								
**5. Network crafting T1**	3.62	0.81	0.03	0.10	0.02	0.21**	0.64						
**6. Positive affect T2**	3.62	0.91	−0.08	0.05	−0.01	0.06	0.45**	0.61					
**7. Information exchange T2**	3.75	0.89	0.03	0.01	0.17*	0.22**	0.62**	0.43**	0.81				
**8. Work-to-family facilitation T3**	3.73	0.81	−0.01	−0.01	0.01	0.17*	0.66**	0.55**	0.64**	0.77			
**9. Work goal attainment T3**	4.68	1.02	−0.03	0.05	0.03	0.15*	0.59**	0.43**	0.64**	0.62**			
**10. Goal difficulty T3**	2.71	0.69	−0.04	0.09	−0.03	0.15*	0.39**	0.31**	0.29**	0.30**	0.29**		
**11. Goal time spending T3**	3.25	1.28	0.19**	0.06	0.05	0.24**	0.14**	0.10*	0.12**	0.10*	0.17**	0.13**	

The value of the diagonal of the table represents AVE scores. *p < 0.05; **p < 0.01; *N* = 199 participants.

We further examined the measurement model by comparing several alternative measurement models and choosing the best fitting one. We recognized four latent constructs (i.e., network crafting, positive affect, information exchange, and work-to-family facilitation) and one observed construct (i.e., goal attainment, measured by a single item). For the single measurement, we specified it by fixing its factor loading to 1 and fixing its error term to 0 (see Hayduk and Littvay, 2012). According to the results (see Table 2), the best fitting model was Model 1, which includes 5 distinct constructs – network crafting, positive affect, information exchange, work-to-family facilitation, and goal attainment: Chi-square = 214.658, d.f. = 109, CFI = 0.944, TLI = 0.930, RMSEA = 0.070, and SRMR = 0.052. This result confirmed the construct discriminant validity. We also tested alternative measurement models and compared them by the Chi-square difference test. The results showed that the five-factor model was significantly better than all the alternative measurement models. Due to the page limits, we presented these results in Table 2.

**Table 2:** Results of measurement models and chi-square difference test.

Models	*χ* ^2^	df	RMSEA	CFI	TLI	SRMR	Compare mode differences
1. Five-factor model*	214.658	109	0.070	0.944	0.930	0.052	
2. Four-factor model (a)	594.324	116	0.144	0.746	0.702	0.110	Model 1 vs. Model 2: Δχ^2^ (7) = 379.666, *p* < 0.001
3. Four-factor model (b)	396.565	129	0.102	0.863	0.838	0.071	Model 1 vs. Model 3: Δχ^2^ (20) = 181.907, *p* < 0.001
4. Three-factor model	627.746	132	0.137	0.746	0.706	0.110	Model 1 vs. Model 4: Δχ^2^ (23) = 413.088, *p* < 0.001

Five-factor model includes: network crafting, positive affect, information exchange, work goal attainment, and work-to-family facilitation; Four-factor model (a) includes: collapsing positive affect and information exchange into one factor; Four-factor model (b) includes: collapsing work goal attainment and work-to-family facilitation into one factor; Three-factor model includes: collapsing positive affect and information exchange into one factor as well as collapsing work goal attainment and work-to-family facilitation into one factor. * refers to the best fitting model.

### Hypothesis testing

**Figure 2:**
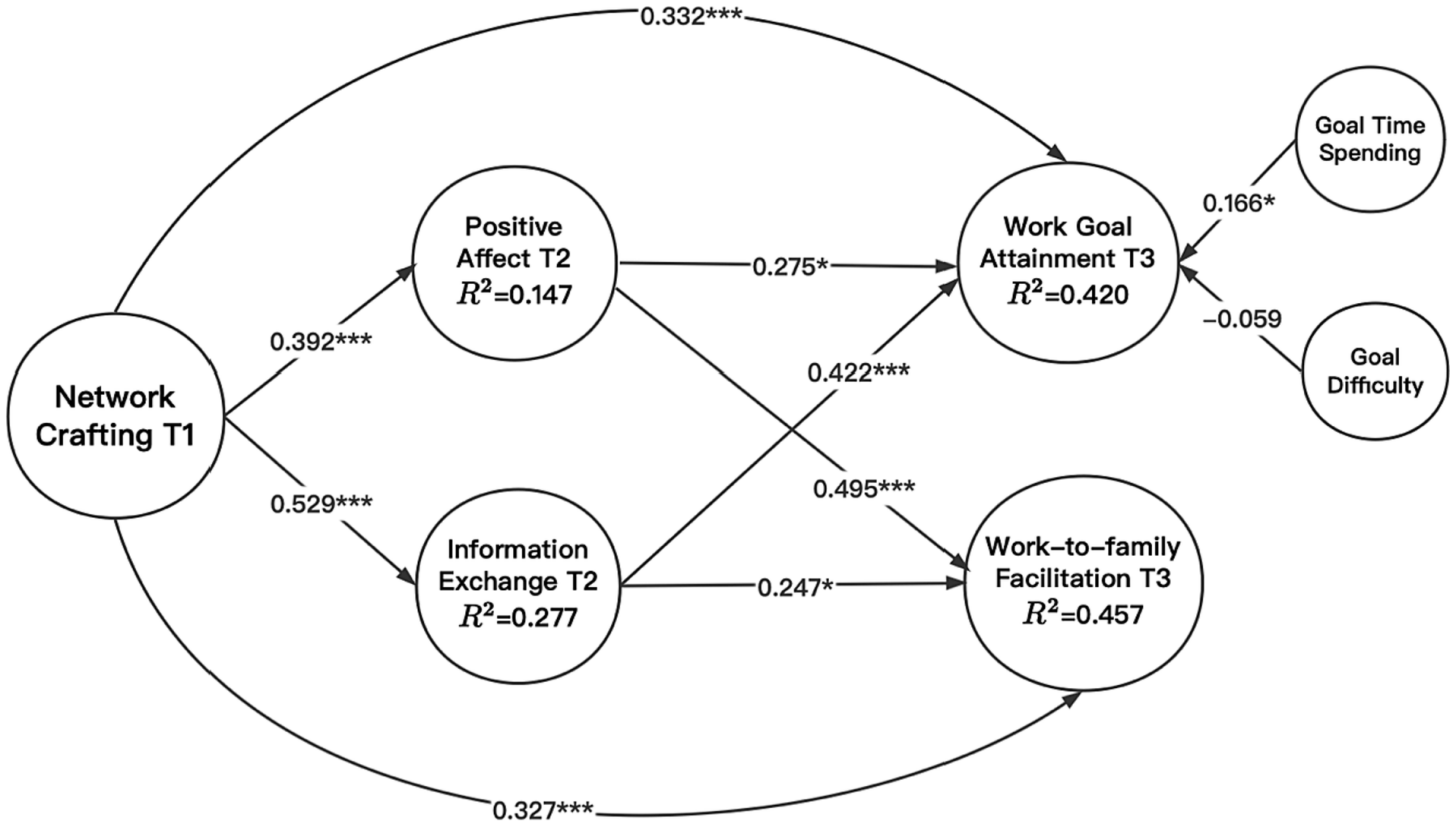
Visualizing coefficients and R square in the plotted model. ***p < 0.001; **p < 0.01; *p < 0.05; *N* = 199.

Based on the best-fitting measurement model, we drew the structural model by estimating the coefficients of each path and R square. The results in Figure 2 showed that R square for positive affect, information exchange, goal attainment, and work-to-family facilitation was 0.147, 0.277, 0.420, and 0.457 respectively, which showed good model fit for the structural model. The results in Table 3 showed that network crafting was positively related to work goal attainment (*b* = 0.332; *p* < 0.001) and that network crafting was positively related to work-to-family facilitation (*b* = 0.327; *p* < 0.001). Hence, H1 and H2 were supported.

**Table 3:** Results of the path analysis (*N* = 199).

We controlled for age, gender, tenure, and educational degree	Estimate	s.e.	t-statistics	value of *p*
Network crafting T1 - > information exchange T2	0.529	0.052	10.052	0.000
Network crafting T1 - > positive affect T2	0.392	0.057	6.709	0.000
Network crafting T1 - > work-to-family facilitation T3	0.327	0.053	6.061	0.000
Network crafting T1 - > work goal attainment T3	0.332	0.047	6.941	0.000
Information exchange T2 - > work-to-family facilitation T3	0.247	0.119	2.179	0.029
Information exchange T2 - > work goal attainment T3	0.422	0.092	4.679	0.000
Positive affect T2 - > work-to-family facilitation T3	0.495	0.131	3.687	0.000
Positive affect T2 - > work goal attainment T3	0.275	0.105	2.502	0.012
Goal difficulty T3 - > work goal attainment T3	−0.059	0.082	0.726	0.468
Goal time spending T3 - > work goal attainment T3	0.166	0.068	2.489	0.013
Network crafting T1 - > information exchange T2 - > work-to-family facilitation T3	0.131	0.065	2.082	0.037
Network crafting T1 - > information exchange T2 - > work goal attainment T3	0.223	0.055	4.134	0.000
Network crafting T1 - > positive affect T2 - > work-to-family facilitation T3	0.196	0.066	2.805	0.005
Network crafting T1 - > positive affect T2 - > work goal attainment T3	0.109	0.048	2.104	0.035

In support of Hypothesis 3, we found that the indirect effect of network crafting on work goal attainment via positive affect was *b* = 0.109, *p* = 0.035, after controlling for goal time spending and goal difficulty; the indirect effect of network crafting on work-to-family facilitation via positive affect was *b* = 0.196, *p* = 0.005. Thus, H3 was supported.

Finally, we found that the indirect effect of network crafting on work goal attainment via information exchange was *b* = 0.223, *p* < 0.001, after controlling for goal time spending and goal difficulty; the indirect effect of network crafting on work-to-family facilitation via information exchange was *b* = 0.131, *p* = 0.037. In conclusion, H4 was supported.

## Discussion

Based on the job crafting literature, broaden-and-build theory, and social cognitive theory, the current study examined the beneficial role of network crafting behaviors on hotel employees’ work and non-work outcomes. Using a three-wave survey design of 199 hotel employees, we revealed a dual process of network crafting. The results of the structural equation modeling showed that network crafting was positively associated with work goal attainment and work-to-family facilitation through increasing positive affect, and that network crafting can also positively relate to work goal attainment and work-to-family facilitation through increasing information exchange.

Specifically, our main effect results (i.e., H1 and H2) imply that network crafting behaviors have positive impacts on employee work/nonwork outcomes. This result aligns with the mainstream job crafting literature (see reviews Zhang and Parker, 2019; Wang et al., 2020) indicating that those who take the initiative to adjust their own job conditions can yield beneficial outcomes, such as work engagement, person-job fit, and job satisfaction. This finding is also in line with previous networking studies revealing that networking-related behaviors can improve employee performance (Porter and Woo, 2015), career advancement (Spurk et al., 2015), and work-nonwork enrichment (Baumeler et al., 2018).

Our mediation effect results (i.e., H3 and H4) reveal by what means network crafting facilitates employee work/nonwork outcomes. We indicate two mediating processes: an affective process (i.e., positive affect) and a social cognitive process (i.e., information exchange). Thus, we echo the broaden-and-build perspective highlighting the beneficial effect of positive emotions and the spillover effect of positive emotions cross domains (Fredrickson, 2004). This finding is in line with the study of Baumeler et al. (2018) showing that networking behaviors can increase work-nonwork enrichment through positive affect at work. Besides, we echo the social cognitive perspective (Bandura, 1989; Patrick et al., 1999) by highlighting the mediating role of information exchange. We indicate that network crafting is able to enhance social learning and resource exchanging and sharing, which will enrich both work domain and family domain. This aligns with the study of Gong et al. (2012) emphasizing that a proactive process at work is able to boost information exchange and psychological safety.

### Theoretical implications

First, the current study adds to the social network and networking literature (Seibert et al., 2001; Carpenter et al., 2012; Porter and Woo, 2015) by suggesting a network-related work strategy – network crafting. Previous studies indicated that social networks are vital to the creative process, career, and trust (Wu, 2008; Baer, 2010; Carpenter et al., 2012). But researchers still call for a better understanding of how to effectively capitalize on the benefits of the social network (Porter and Woo, 2015). Although some recent studies emphasized that expansive-oriented networking behaviors, such as making, using, and maintaining internal and external contacts, can tap into valuable social capital and improve work and career outcomes (Wolff and Moser, 2009, 2010), we may miss an optimization-oriented perspective to understand the effective networking process. Thus, incorporating the job crafting literature (Wrzesniewski and Dutton, 2001; Tims et al., 2012), our study aims to examine the beneficial role of network crafting to provide insights into how employees can smartly network – that is, not only seeking resources from the social network but also optimizing demands derived that drain one’s energy, time, and effort. Our results showed that network crafting behavior was positively related to both work and non-work outcomes (i.e., work goal attainment and work-to-family facilitation). This implies that an optimization-oriented network strategy is effective and helpful to the goal attainment process and work-family enrichment process.

Second, we enrich the existing job crafting and job (re)design literature (Wrzesniewski and Dutton, 2001; Tims et al., 2012; Oldham and Fried, 2016). Plenty of studies have indicated the beneficial role of job crafting on employee various outcomes (see meta-analyses Rudolph et al., 2017; Lichtenthaler and Fischbach, 2019; Zhang and Parker, 2019). Other studies suggest that employee bottom-up job redesign approaches, such as playful work design (Scharp et al., 2019), strengths use (Bakker and van Wingerden, 2021), and proactive vitality management (Bakker et al., 2020), are beneficial to work outcomes (e.g., work engagement and creativity). Our study enriches this line of research and adds network crafting behavior as another effective job redesign strategy for employees. We highlight that to better redesign jobs and increase person-job fit, employees can particularly consider crafting their networks, in addition to crafting their job conditions in general. This is because, as our results showed, network crafting can effectively activate a positive affect process and an information exchange process (i.e., the mediating roles of positive affect and information exchange).

Third, our study also contributes to the work-family enrichment literature (Wayne et al., 2007). Work-family researchers (Frone, 2003; Greenhaus et al., 2006) have provided many insights into the ways of improving work-to-family facilitation, such as increasing personal resources (e.g., self-efficacy; Ten Brummelhuis and Bakker, 2012; Gashi Tresi and Mihelič, 2018), job resources (e.g., social support from colleagues and leaders; Hakanen et al., 2011), and personal actions (e.g., work reflection, job crafting; Wayne et al., 2007; Rastogi and Chaudhary, 2018). Our study further enriches work-family facilitation studies by highlighting that network crafting is also a meaningful approach to transmitting and sustaining employee positive work experiences and practices into their family domain. By examining the mediating roles of positive affect and information exchange, we revealed a dual process of network crafting on work-to-family facilitation. That is, network crafters can facilitate the work-to-family process by experiencing more positive affect and exchanging more useful information with others. This result is also in line with the resource-gain-development perspective (Wayne et al., 2007) suggesting that personal developmental gains (e.g., new information and perspectives) and affective gains (e.g., positive emotion) in one domain can enhance the functioning of the other domain. In our study, network crafting was found to be effective in obtaining developmental gains and affective gains. Our mediation findings also align with the work-family enrichment model (Greenhaus et al., 2006) demonstrating that the work domain can enrich the family domain via an affective path and an instrumental path.

Fourth, our study also adds to a better understanding of the social cognitive theory and broaden-and-build theory (Bandura, 1989; Fredrickson, 2004) in the hospitality context. The post-pandemic era has brought about new and diverse job demands for hotel workers. Our findings shed light on how hotel workers can effectively engage in network crafting behaviors to facilitate the broaden-and-build process and social learning process. By incorporating insights from the crafting literature (Rudolph et al., 2017; Wang et al., 2020), we emphasize the significance of proactively shaping professional networks as a crucial factor in stimulating the broaden-and-build process, which fosters the development of positive emotions, as well as the social learning process, which facilitates information exchange. We emphasize that these two psychological processes are essential for the growth and development of hotel employees, leading to positive outcomes both in their work and personal lives.

Finally, we enrich the hospitality management studies (Xu and Chris Cao, 2019; Liu et al., 2022). We contextualized our sample in the hotel industry with an exclusive focus on hotel employees. Hospitality management researchers emphasize hospitality workers have high work overload, inflexible work schedules, less holidays, and work-family interference (Liu et al., 2022; Pan and Li, 2022). It is important to seek ways to help hospitality workers to manage their work overload and increase work-family balance. Thus, our study provided valuable insights into how hotel employees can smartly network (i.e., seeking resources from networks and optimizing hindering demands that are derived from some contacts), and in turn, improve their work goal attainment and work-to-family facilitation. Previous hospitality management studies revealed many factors that can contribute to hospitality workers’ work/non-work outcomes, such as inclusive HR practices (Gehrels and Suleri, 2016), servant leadership (Liu et al., 2022), core self-evaluations (Lee and Ok, 2015), deep acting of emotional labor (Lee and Madera, 2019), and job crafting (Teng, 2019). We enrich this line of research and add network crafting as another effective strategy to help hotel employees to achieve work goals in the work domain and transmit these positive gains into the family domain.

### Practical implications

Our study provides several practical implications for hotel employees and hotel managers. First, we suggest hotel employees should take a deeper look at their professional networks, analyze their personal needs and goals, and then proactively shape their networks as a whole, gaining benefits and optimizing potential threats. This is what we call a network crafting process. Our study has shown the beneficial role of network crafting on work goal attainment and work-to-family facilitation. Therefore, we also suggest that hotel employees should hold a mindset on network crafting. When they feel the need to solve work-related problems, they can realize that they can make optimal use of their networks to find meaningful solutions to the problems (van Gool, 2022). Specifically, employees can actively seek opportunities to connect with colleagues, industry professionals, mentors, and leaders within their organization or field. This can be done through attending networking events, joining professional associations, participating in industry conferences, or engaging in online networking platforms. Building and maintaining relationships is also a crucial process for network crafting. Employees can invest time and effort in nurturing relationships by regularly connecting with their network contacts, offering assistance or support when needed, and expressing genuine interest in others’ professional development and success. This can involve providing assistance, sharing resources, or collaborating on projects or initiatives. Finally, employees can leverage social media and online Platforms to craft networks. In today’s digital age, employees can utilize social media platforms, such as LinkedIn, WeChat, or industry-specific online forums to connect with professionals, join relevant groups or communities, and engage in discussions and knowledge sharing.

Second, from the perspective of hotel managers, we suggest that managers should empower employees to engage in network crafting behavior (Zhang and Parker, 2019). Hotel managers can consider giving employees autonomy to shape their network conditions, so that they feel in control of their tasks and relations. It is also wise for managers to clearly demonstrate the organizational goals and encourage employees to craft their networks based on organizational goals (Wrzesniewski and Dutton, 2001). As such, employees can craft their networks toward shared goals and interests, which will not become an individual-focused behavior but also a collective-focused behavior. This can benefit organizational performance and thriving. Specifically, leaders can serve as role models by demonstrating effective networking behaviors themselves. By openly sharing their own networking experiences and actively engaging in networking activities, leaders can inspire employees to follow suit and feel more comfortable in expanding their own professional networks. Leaders can also offer training sessions or workshops focused on specific network crafting skills and techniques. These sessions can provide employees with practical tips, strategies, and best practices for effective network crafting. Additionally, leaders can share resources such as books, articles, or online courses to help employees develop their network crafting capabilities.

### Limitation and future directions

We also acknowledge some limitations in this study, which can represent future research directions. First, all constructs we measured were self-reported. Although we examined the common methods bias and it was not an issue, we still recommend future studies to use multiple source raters to measure employees’ work/non-work outcomes. For example, future studies can use leader-rating to evaluate employee work outcomes and use family partner-rating to evaluate employee family outcomes. Second, we are not sure if our findings can be generalized to other cultural backgrounds or other country settings. China has a different cultural background compared to western cultures (Hofstede and Bond, 1988). Future studies can replicate our model in other cultural settings to provide more nuanced insights into hotel employees’ network crafting process. Besides, we are not sure if the three hotels in Jinan can be a representative for the Chinese sample, as we just used a convenient sampling approach. We recognized it as a limitation of this study and touched upon this issue as a valid future research direction. Third, we did not examine any potential moderators in our model. It is very possible that the network crafting process may vary from different personalities and contextual factors (e.g., leadership, group norm, and/or organizational climate and policy). It will be promising to investigate these potential moderators in future studies. As such, we can gain insights into when (i.e., under what conditions) network crafting can become more effective. Further, it is even more meaningful for future studies to develop effective interventions to train hotel employees to master network crafting behaviors and apply them in their real work and family life.

## Conclusion

To conclude, using a three-wave survey of 199 hotel employees, we proposed and examined the beneficial role of network crafting on hotel employees’ work/non-work outcomes. Using the structural equation modeling approach, we revealed a dual process of network crafting. That is, network crafting can positively link to work goal attainment and work-to-family facilitation through increasing positive affect and information exchange. Thus, we provide insights into how network crafting can boost an affective process and an information exchange process. We provide a means for hotel employees to effectively manage their work and family life. We highlight that it is important for hotel employees and managers to recognize the value of network crafting and actively engage in this strategy in daily life.

## Data availability statement

The raw data supporting the conclusions of this article will be made available by the authors, without undue reservation.

## Ethics statement

The studies involving humans were approved by Lingnan University Ethics Committee. The studies were conducted in accordance with the local legislation and institutional requirements. The participants provided their written informed consent to participate in this study.

## Author contributions

HZ: Conceptualization, Data curation, Investigation, Project administration, Resources, Writing – original draft. JL: Conceptualization, Investigation, Resources, Writing – review & editing. HW: Conceptualization, Writing – review & editing, Methodology, Supervision. KL: Methodology, Software, Writing – review & editing.
